# Association between the hemoglobin-to-red cell distribution width ratio and three-month unfavorable outcome in older acute ischemic stroke patients: a prospective study

**DOI:** 10.3389/fneur.2025.1534564

**Published:** 2025-03-11

**Authors:** Luwen Huang, Linlin Li, Qing-rong Ouyang, Ping Chen, Ming Yu, Lei Xu

**Affiliations:** ^1^Department of Neurology, Suining Central Hospital, Suining, Sichuan Province, China; ^2^Department of Pharmacy, Suining Central Hospital, Suining, Sichuan Province, China

**Keywords:** acute ischemic stroke, hemoglobin-to-red cell distribution width ratio, prognosis, association, older participants

## Abstract

**Objective:**

Acute ischemic stroke (AIS) is a prevalent acute condition among older individuals. This study is the first investigation of the link between the HRR and unfavorable three-month outcome in older AIS patients.

**Methods:**

This secondary research used data from a sample of 1,470 older AIS patients collected from a South Korean hospital between January 2010 and December 2016. Multiple imputation was applied to account for absent values. Binary logistic regression analysis was used to examine the relationship between the baseline HRR and adverse outcome at three-month. Restricted cubic spline analysis was employed to evaluate the correlation between HRR levels and adverse outcome. Interaction tests were performed to discern variations among subgroups.

**Results:**

At 3 months, the overall incidence of adverse events was 31.43%, with a median HRR of 9.49. Compared to those with a lower HRR (Q1), the adjusted odds ratios (ORs) for the HRR in Q2, Q3, and Q4 were 0.61 (95% CI: 0.41–0.92, *p* = 0.017), 0.49 (95% CI: 0.31–0.78, *p* = 0.003), and 0.54 (95% CI: 0.31–0.92, *p* = 0.025), respectively. The correlation between the HRR and adverse outcome was non-linear (*p* < 0.05). An inflection point threshold of 10.70 was established via RCS analysis. Each 1-unit increase in HRR on the left side of the infection point was associated with a 24.0% decrease in the likelihood of adverse outcomes (OR = 0.76, 95% CI: 0.66–0.86, *p* < 0.001). ROC analysis revealed that HRR had the highest AUC (0.64, 95% CI: 0.61–0.67), followed by hs-CRP (0.60, 95% CI: 0.57–0.63), FPG/HbA1c (0.59, 95% CI: 0.55–0.63), and WBC (0.55, 95% CI: 0.51–0.58).

**Conclusion:**

A lower HRR was correlated with a higher risk for adverse outcome in older AIS patients.

## 1 Introduction

Stroke is the second leading cause of mortality worldwide and the third major contributor to disability in non-communicable diseases; acute ischemic stroke (AIS) constituted approximately 62.4 to 67.7% of all stroke incidents in 2021 (1). AIS is a common illness among the older population. Between 1990 and 2019, the prevalence of ischemic stroke among older adults was markedly greater than that among younger adults worldwide (2). Consequently, it is imperative to determine appropriate and effective clinical indicators to predict AIS prognosis in geriatric patients, guide clinical care, and improve treatment outcome.

Red blood cell distribution width (RDW), which reflects the variability in red blood cell volume, has traditionally been used for the diagnosis and differential diagnosis of anemia (3). Clinical studies have demonstrated that RDW is increasingly acknowledged as an independent risk factor for recurrence, hemorrhagic transformation, in-hospital mortality, and poststroke fatigue in patients with AIS (4–7). Moreover, recent research has identified RDW as a potential inflammatory marker significantly associated with stroke-associated pneumonia (SAP) and as a valuable tool for enhancing SAP risk stratification in thrombolyzed AIS patients when integrated into established prediction models (8). Nevertheless, a study including 1,504 patients indicated that RDW could not predict the severity or functional results of AIS (9). Therefore, novel and dependable markers are needed to predict AIS outcome. These limitations underscore the urgent need for novel, dependable biomarkers to improve AIS outcome prediction.

The hemoglobin-to-red blood cell distribution width ratio (HRR) is a novel biomarker first introduced by Peng et al. in their research on the progression of esophageal squamous cell cancer (10). HRR has demonstrated a strong correlation with inflammatory levels and has been associated with adverse outcomes in various diseases (10–16). Compared to single inflammatory markers such as WBC or hs-CRP, HRR offers a unique advantage by simultaneously reflecting red blood cell metabolism and systemic inflammation. As a simple and easily obtainable parameter, HRR may provide a more comprehensive prediction of unfavorable outcomes in AIS patients. Importantly, studies have also shown a negative association between HRR and poor outcome in AIS patients (17–19).

Nonetheless, the correlation between HRR and negative outcome in elderly AIS patients remains unclear. This study aimed to address this gap by investigating the correlation between HRR and unfavorable outcome. The ultimate goal is to establish HRR as a simple and accessible biomarker that can aid clinicians in early risk stratification, thereby improving patient management and enhancing quality of life.

## 2 Materials and methods

### 2.1 Data sources

This research performed a secondary analysis of a prospective cohort study. Data were gathered from January 2010 to December 2016 through a single-center prospective registry in South Korea (20). The research was approved by the Institutional Review Board of Seoul National University Hospital (IRB No. 1009–062-332, 20). All data were anonymized to protect patient privacy, and the requirement for informed consent was waived by the board. The study methodology conformed to the standards set by the Declaration of Helsinki. The following data were acquired from the subsequent study: Kang et al. (20). The material is freely available and may be used, disseminated, and reproduced in any format, depending on appropriate attribution to the original authors and source. This is authorized under the Creative Commons Attribution License (20).

### 2.2 Study population

**Figure 1:**
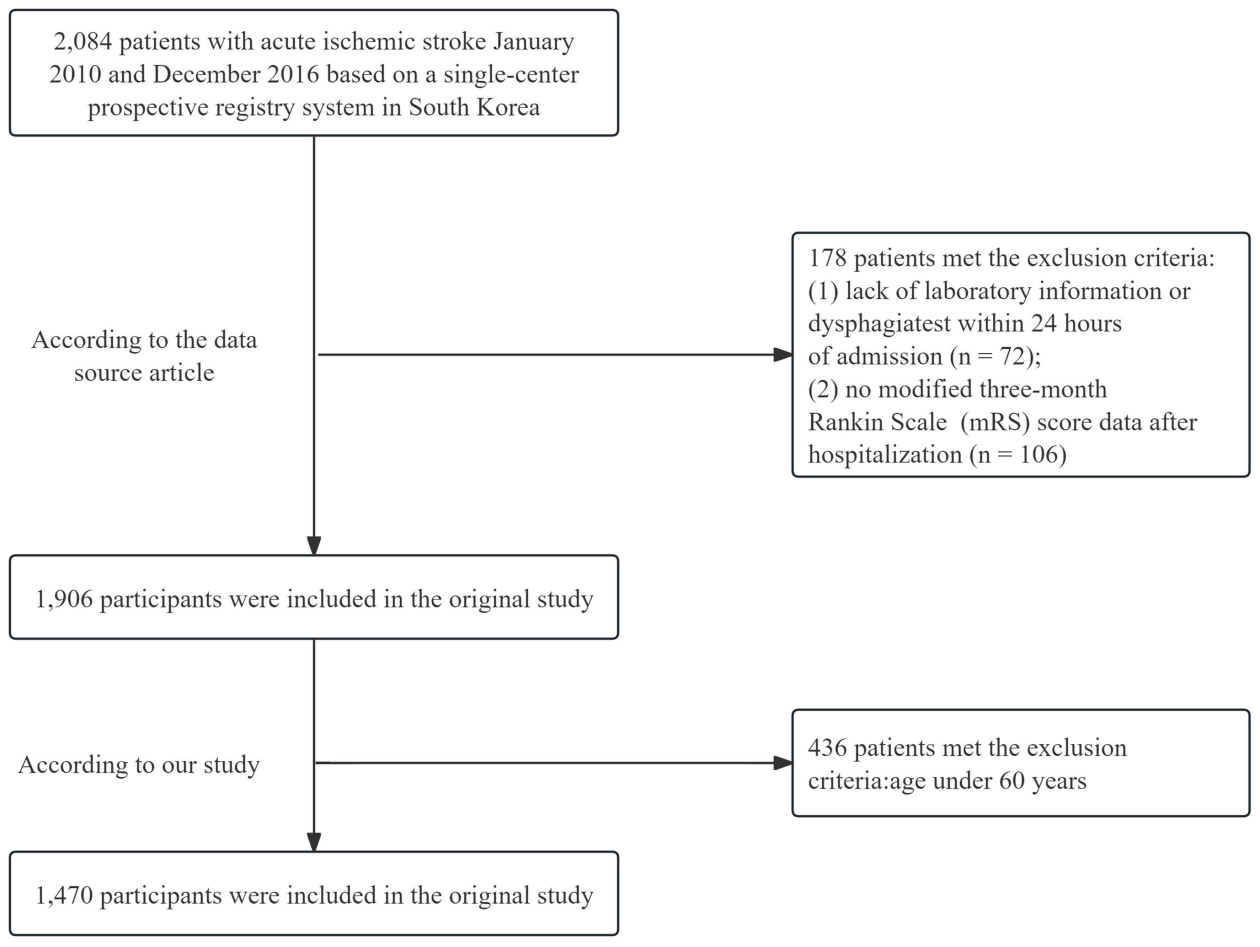
Flow chart.

The initial trial included 2,084 individuals with AIS admitted within 7 days of initial symptoms, based on a prospective registry approach (20). The exclusion criteria were as follows: (1) absence of laboratory data or dysphagia assessment within 24 h of admission; (2) lack of modified 3-month Rankin scale (mRS) score information posthospitalization; and (3) age under 60 years. In total, 1,470 patients were included in this study (Figure 1). Patients without dysphagia assessment within 24 h were excluded to prevent bias, as early dysphagia evaluation is crucial for preventing complications and improving prognosis in ischemic stroke (21).

### 2.3 Variables and covariates

The HRR was calculated by dividing hemoglobin (Hb; g/L) by the red cell distribution width (RDW; %) (17, 18). The covariates included the following: (1) continuous variables such as white blood cell count (WBC), red blood cell count (RBC), hemoglobin (Hb), red cell distribution width (RDW), platelet count (PLT), low-density lipoprotein cholesterol (LDL-C), blood urea nitrogen (BUN), serum creatinine (Scr), alanine aminotransferase (ALT), fasting blood glucose (FBG), hemoglobin A1c (HbA1c), high-sensitivity C-reactive protein (hs-CRP), fibrinogen (FIB), and body mass index (BMI) (20). (2) The categorical factors included sex, age, hypertension, diabetes mellitus (DM), hyperlipidemia, smoking status, atrial fibrillation (AF), coronary heart disease (CHD), National Institutes of Health Stroke Scale (NIHSS) score, and stroke etiology. Laboratory data collected within 24 h of admission were retrieved from electronic healthcare records (20). Body mass index (BMI) was calculated by dividing weight in kilograms by the square of height in meters (kg/m^2^). Stress hyperglycemia was evaluated using the following formula: FBG (mg/dl)/HbA1c (%).

### 2.4 Endpoints

The primary outcome was characterized as an unfavorable AIS result after 3 months, evaluated using the mRS score (22). Patient follow-up was primarily conducted through outpatient visits or structured telephone interviews, both performed by neurologists (20). An unfavorable outcome is defined by an mRS score of 3 or higher, while a favorable outcome is defined by an mRS score of 2 or lower (22).

### 2.5 Statistical analysis

Continuous variables are expressed as the mean ± standard deviation (SD) or median (interquartile range). Student’s t-test or the Mann–Whitney U test was utilized on the basis of the normality of the distribution. Categorical variables are expressed as counts (%), and the chi-square test (or Fisher’s exact test) was employed to examine differences among the four HRR quartiles (23).

Binary logistic regression analyses, both univariate and multivariate, were conducted to minimize the influence of different variables on adverse outcomes. Confounders were determined according to the following criteria: (1) The factor exerted a substantial influence (>10%) on the research variable. (2) Certain factors had a documented influence on the outcome variable according to previous studies (17, 18). (3) In the univariate analysis, factors with a *p*-value less than 0.05 were deemed significant. In multivariate analysis, various statistical models are employed to guarantee the stability of the results. No variables were adjusted in the crude model. Model I was adjusted for age and sex, while Model II included adjustments for 17 additional variables: WBC, RBC, PLT, LDL-C, BUN, FBG, ALT, hs-CRP, BMI, hypertension, DM, hyperlipidemia, CHD, AF, smoking status, NIHSS score at admission, and previous mRs. To control for the false discovery rate (FDR) in multiple hypothesis testing, we applied the Benjamini-Hochberg (BH) procedure.

Restricted cubic spline (RCS) analysis was employed to evaluate the potential nonlinear association between the HRR and adverse outcome. If a non-linear association was identified, a two-piecewise regression model would be conducted to calculate the threshold effect of HRR on poor outcomes, based on the smoothing plot. The HRR turning point was determined via exploratory analysis, wherein trial turning points were evaluated over predefined intervals, ultimately selecting the one with the highest model likelihood. A log-likelihood ratio test (LRT) was employed to identify the optimal model characterizing the association between the HRR and 3-month adverse outcome in senior AIS patients (24).

Furthermore, interaction and stratified analyses were conducted based on age, sex, hypertension status, DM status, hyperlipidemia status, smoking status, CHD status, BMI, and NIHSS score upon admission. Missing values were addressed by multiple imputations. Information on the missing data is shown in Supplementary Table 1. The area under the receiver operating characteristic (ROC) curve (AUC) and the corresponding 95% confidence intervals (CI) were calculated to evaluate and compare the predictive performance of HRR, hs-CRP, FPG/HbA1c, and WBC for unfavorable outcomes in older adults with AIS.

The data were analyzed via R (The R Foundation; version 4.2.0)^1^ and EmpowerStats (X&Y Solutions, Inc., Boston, MA)^2^ (24). A two-sided *p* value below 0.05 was considered statistically significant.

## 3 Results

### 3.1 Baseline characteristics of the study patients

Following screening, 1,470 population with AIS were included in the data analysis. The baseline characteristics of the population individuals by HRR quartiles are presented in Table 1 (Q1: <8.96, Q2: 8.96–10.30, Q3: 10.31–11.30, and Q4: >11.30). The participants were categorized into the following age groups: 60 to <70 years (*n* = 505, 34.35%); 70 to <80 years (*n* = 670, 45.58%); and ≥ 80 years (*n* = 295, 20.07%). Supplementary Table 2 presents the demographics, laboratory variables, comorbidities, and other pertinent data categorized by HRR quartiles. Supplementary Table 2 illustrates notable disparities in age; sex; WBC, RBC, Hb, RDW, LDL-C, BUN, Scr, ALT, FBG, and hs-CRP levels; BMI; DM; smoking status; the National Institutes of Health Stroke Scale (NIHSS) score at admission; and stroke etiology.

**Table 1:** Relationship between HRR and unfavorable outcome 3 months after AIS in different models.

Variable	Crude model (OR, 95%CI)	*p-*value	Model I (OR, 95%CI)	*p-*value	Model II (OR, 95%CI)	*p-*value
HRR	0.82 (0.77, 0.87)	<0.001*	0.85 (0.80, 0.91)	<0.001*	0.81 (0.72, 0.91)	<0.001*
HRR (Quartiles)						
Q1 (<8.96)	Ref		Ref		Ref	
Q2 (8.96–10.30)	0.59 (0.44, 0.80)	<0.001*	0.59 (0.44, 0.81)	<0.001*	0.65 (0.44, 0.97)	0.028*
Q3 (10.30–11.30)	0.43 (0.31, 0.59)	<0.001*	0.51 (0.37, 0.70)	<0.001*	0.53 (0.33, 0.84)	0.007*
Q4 (>11.3)	0.43 (0.32, 0.59)	<0.001*	0.57 (0.41, 0.79)	<0.001*	0.58 (0.34, 0.96)	0.039*
*p* for trend	0.81 (0.75, 0.87)	<0.001*	0.86 (0.80, 0.92)	<0.001*	0.87 (0.76, 0.98)	0.024*

Crude mode1: we did not adjust for other covariates; Model I: we adjusted for sex and age; Model II: Model I + WBC, RBC, PLT, LDL-C, BUN, FBG, ALT, hs-CRP, BMI, hypertension, DM, hyperlipidemia, CHD, AF, smoking status, NIHSS score at admission, and previous mRs. *Indicates statistical significance after Benjamini-Hochberg.

Supplementary Table 3 indicates that 462 participants experienced unfavorable consequences. The group with poor outcome at 3 months presented a decreased HRR (mean: 9.49 vs. 10.23, *p* < 0.001). Univariate analysis indicated that age, sex, WBC, RDW, BUN, LDL-C, FBG, hs-CRP, hypertension, DM, and AF were correlated with worse outcome (all *p* < 0.05; Supplementary Table 4). Adverse outcome in AIS patients were strongly correlated with an undetermined cause (OR = 2.46, 95% CI: 1.58–3.82; *p* < 0.001).

### 3.2 Associations between the baseline HRR and unfavorable outcome

Table 1 displays the unadjusted and multivariable-adjusted correlations between the HRR and adverse outcome. Analysis of the HRR as a continuous variable revealed an inverse correlation with adverse outcome (non-adjusted model: OR = 0.82, 95% CI: 0.77–0.87, *p* < 0.001; Model I: OR = 0.85, 95% CI: 0.80–0.91, *p* < 0.001; Model II: OR = 0.80, 95% CI: 0.71–0.90, *p* < 0.001). The likelihood of adverse outcome in AIS patients decreased with each 1-unit increase in the HRR. Additionally, when the HRR was examined as a categorical variable, patients with decreased HRR levels had a markedly elevated risk of adverse outcome in AIS patients. In comparison to the reference group (Q1), the adjusted ORs for participants in Q2, Q3, and Q4 were 0.61 (95% CI: 0.41–0.92, *p* = 0.017), 0.49 (95% CI: 0.31–0.78, *p* = 0.003), and 0.54 (95% CI: 0.31–0.92, *p* = 0.025), respectively (*p* for trend = 0.01). After employing multiple imputations to address missing data, the results remained consistent with prior findings (Table 2). After applying the Benjamini-Hochberg correction to control for false discovery rate, all tests for HRR (both continuous and categorical variables), including the HRR quartiles (Q2, Q3, Q4) and the trend test, remained statistically significant. Sensitivity analysis evaluated the HRR values as continuous and categorical variables before and following multiple imputations, consistently yielding similar results for adverse outcome.

**Table 2:** Relationship between HRR and unfavorable outcome 3 months after AIS in different models by using multiple imputation.

Variable	Crude model (OR, 95%CI)	*p-*value	Model I (OR, 95%CI)	*p-*value	Model II (OR, 95%CI)	*p-*value
HRR	0.82 (0.77, 0.87)	<0.001	0.85 (0.80, 0.91)	<0.001	0.80 (0.71, 0.90)	<0.001
HRR (Quartiles)						
Q1 (<8.96)	Ref		Ref		Ref	
Q2 (8.96–10.30)	0.59 (0.44, 0.80)	<0.001	0.59 (0.44, 0.81)	<0.001	0.65 (0.45, 0.95)	0.026
Q3 (10.30–11.30)	0.43 (0.31, 0.59)	<0.001	0.51 (0.37, 0.70)	<0.001	0.52 (0.34, 0.80)	0.003
Q4 (>11.3)	0.43 (0.32, 0.59)	<0.001	0.57 (0.41, 0.79)	<0.001	0.60 (0.36, 1.00)	0.049
*p* for trend	0.81 (0.75, 0.87)	<0.001	0.86 (0.80, 0.92)	<0.001	0.87 (0.77, 0.98)	0.024

Crude mode 1: we did not adjust for other covariates; Model I: We adjusted for sex and age; Model II: Model I + WBC, RBC, PLT, LDL-C, BUN, FBG, ALT, hs-CRP, BMI, hypertension, DM, hyperlipidemia, CHD, smoking status, and previous mRs.

As shown in Supplementary Table 5, hemoglobin (Hb) was classified into quartiles, and these categorical Hb values were integrated into the model. The multivariate-adjusted model results demonstrated that hemoglobin levels between 134 and 145 g/L were inversely correlated with adverse outcome (OR = 0.58, 95% CI: 0.34–0.99, *p* = 0.047). None of the RDW quartiles exhibited a significant correlation with adverse outcome after controlling for all covariates (Q2: OR = 0.83, 95% CI: 0.56–1.21, *p* = 0.321; Q3: OR = 1.04, 95% CI: 0.71–1.53, *p* = 0.841; Q4: OR = 1.35, 95% CI: 0.91–1.98, *p* = 0.133; Supplementary Table 6).

### 3.3 Analysis of the nonlinear relationship between the baseline HRR and unfavorable outcome

**Figure 2:**
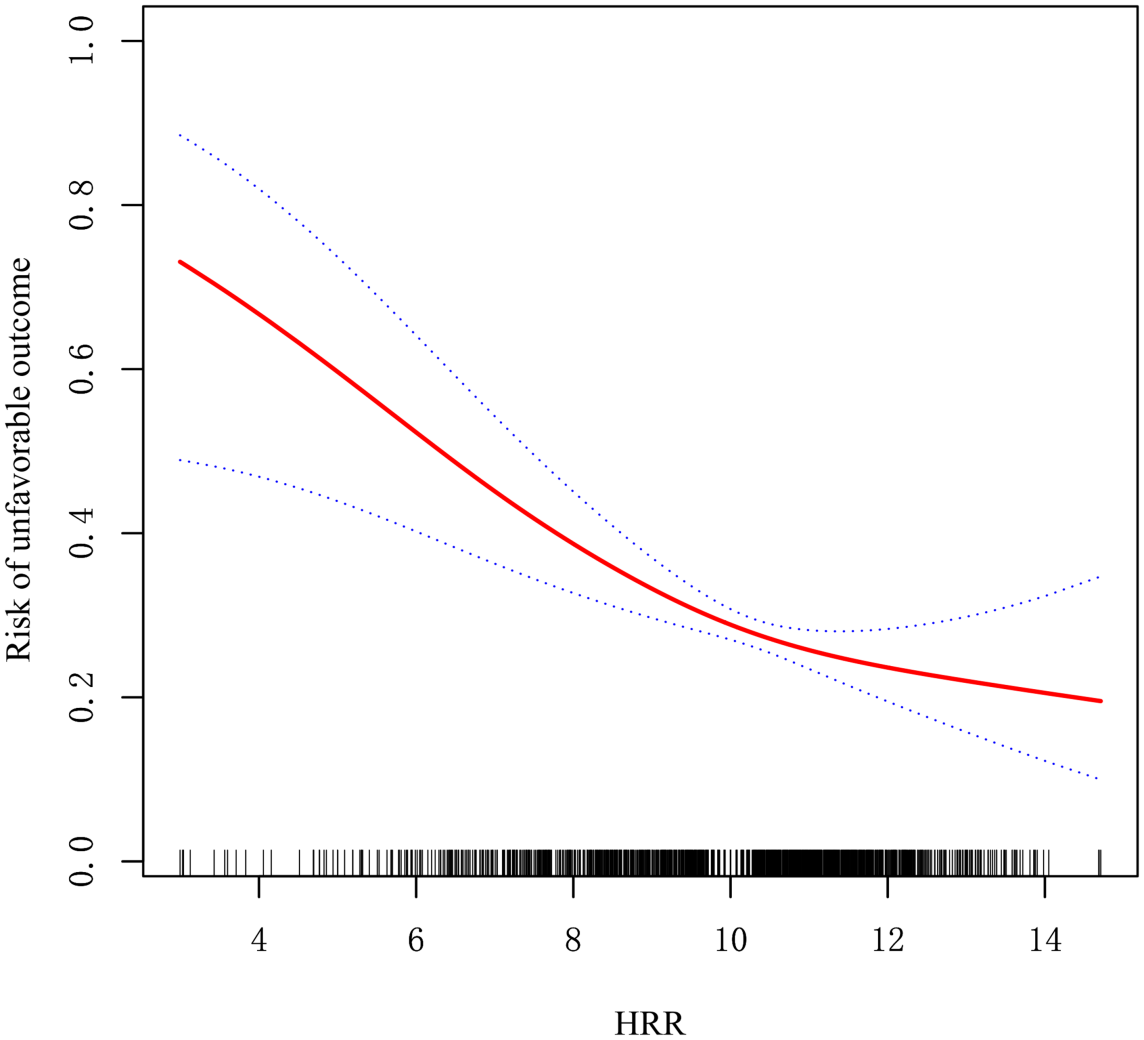
Non-linear relationship observed between the baseline HRR level and the risk of unfavorable outcome. The solid red line represents the smooth curve fit between variables. Blue bands present the 95% confidence interval. The data were adjusted for the variables in Model II.

After adjusting for factors in Model II, a curve-fitting equation for the baseline HRR and adverse outcomes was derived via RCS analysis. We identified a nonlinear correlation between the HRR and adverse outcome (Figure 2). A two-piecewise model was employed in the threshold analysis to evaluate the correlation between the baseline HRR and adverse outcome. The inflection point was determined to be 10.70 (Table 3). To the left of the inflection point, the OR for the HRR was 0.76 (95% CI: 0.66–0.86, *p* < 0.001), indicating a 24% decrease in the likelihood of adverse outcome with each 1-unit increase in the HRR. To the right of the inflection point, the OR was 1.04 (95% CI: 0.82–1.31, *p* = 0.755), indicating that the correlation between the HRR and adverse outcome was not statistically significant when the HRR exceeded 10.71. This finding suggests that the likelihood of adverse outcome decreases with increasing HRR. The *p*-value for the LRT in our research was 0.022.

**Table 3:** Threshold-effect analysis of the relationship between the baseline HRR level and unfavorable outcome 3 months after AIS.

Models	Per-unit increase		
	OR	95%CI	*p-*value
Model I	0.81	0.721–0.91	<0.001
One line effect			
Model II			
Turning point (K)	10.70		
Baseline HRR levels < K	0.76	0.66–0.86	<0.001
Baseline HRR levels > K	1.04	0.82–1.31	0.755
*p*-value for LRT test*			0.022

Model I, one-line linear regression model; Model II, two-piece wise linear regression model. Adjusted for WBC, RBC, PLT, LDL-C, BUN, FBG, ALT, hs-CRP, BMI, hypertension, DM, hyperlipidemia, CHD, AF, smoking status, NIHSS score at admission, and previous mRs. OR, Odd Ratio; CI, confidence interval; LRT, logarithm likelihood ratio test. *p < 0.05 indicates that Model II is significantly different from Model I.

### 3.4 Subgroup analysis

**Figure 3:**
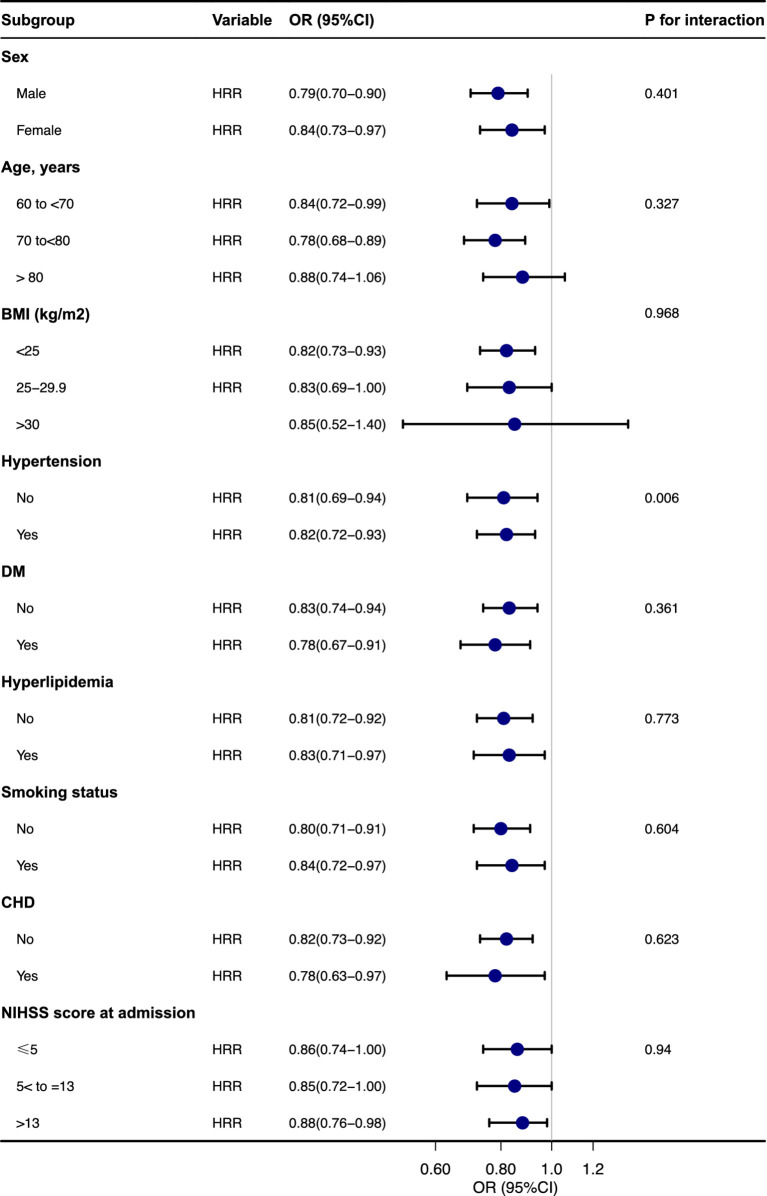
Subgroup analyses of the effect of unfavorable outcomes.

Subgroup analysis was performed to investigate the association between the HRR and unfavorable outcome across multiple variables, including age, sex, hypertension, DM, hyperlipidemia, smoking status, CHD, BMI, and NIHSS score at admission. The findings are illustrated in Figure 3. The analysis of the interaction between the HRR and each subgroup variable indicated no significant interactions (*p* for interaction >0.05).

### 3.5 Comparative predictive value of HRR and other inflammatory biomarkers for 3-month unfavorable outcomes in older AIS patients

In the Crude Model, white blood cell count (WBC) was significantly associated with unfavorable outcomes 3 months after acute ischemic stroke (AIS; OR = 1.07, 95%CI: 1.03–1.11, *p* < 0.001; Supplementary Table 7). This significant association persisted in Model I after adjusting for sex and age (OR = 1.07, 95%CI: 1.03–1.11, *p* = 0.001). However, in the fully adjusted Model II, the association between WBC and unfavorable outcomes was no longer significant (OR = 1.02, 95%CI: 0.97–1.07, *p* = 0.556). Hs-CRP was significantly associated with unfavorable outcomes across all models (Supplementary Table 8). In the Crude Model, each unit increase in hs-CRP was associated with a 14% higher risk of unfavorable outcomes (OR = 1.14, 95%CI: 1.09–1.18, *p* < 0.001). This association remained significant in Model I (OR = 1.12, 95%CI: 1.08–1.17, *p* < 0.001) and Model II (OR = 1.10, 95%CI: 1.05–1.16, *p* < 0.001). For the FPG/HbA1c ratio, a significant association with unfavorable outcomes was observed in the Crude Model (OR = 1.08, 95%CI: 1.05–1.11, p < 0.001) and Model I (OR = 1.09, 95%CI: 1.06–1.12, *p* < 0.001; Supplementary Table 9). However, this association became nonsignificant in the fully adjusted Model II (OR = 1.05, 95%CI: 0.99–1.12, *p* = 0.111).

To further evaluate the predictive value of HRR compared with other inflammatory biomarkers, including hs-CRP, FPG/HbA1c, and WBC, ROC curves and the corresponding AUC values were calculated (Supplementary Figure 1). For unfavorable outcomes within 3 months, the AUCs (95% confidence intervals) were as follows: HRR, 0.64 (0.61–0.67); hs-CRP, 0.60 (0.57–0.63); FPG/HbA1c, 0.59 (0.55–0.63); and WBC, 0.55 (0.51–0.58).

## 4 Discussion

This retrospective observational study investigated the correlation between baseline heart rate recovery (HRR) levels and three-month unfavorable outcome in older patients with AIS, yielding several significant findings. First, the HRR exhibited an inverse relationship with unfavorable outcome after controlling for potential confounders. Second, a threshold effect was identified: when the HRR fell below 10.71, the risk of adverse outcome decreased as the HRR increased. However, in older patients with HRR levels exceeding this threshold, no further reduction in the risk of unfavorable outcome was observed over 3 months. Third, no significant interaction was found between the baseline HRR and adverse outcome in AIS patients, suggesting that the HRR was independently associated with unfavorable outcome across various subgroups. Fourth, the HRR demonstrated better predictive performance for unfavorable outcome. This study bridges the gap in understanding the role of HRR as a composite hematological biomarker derived from Hb and RDW, offering a novel perspective for integrating these routinely available parameters into clinical risk stratification for AIS.

Researchers have proposed several hypotheses to explain why older people with AIS experience negative outcome due to a lower HRR. Initially, elevated RDWs within normal limits may signify enhanced red blood cell breakdown, inefficient erythropoiesis, or a greater quantity of immature red blood cells (25, 26). Concurrently, red blood cells possess significant antioxidant capacity, and abnormalities in these cells might result in diminished cell survival rates (27, 28). Oxidative stress and microcirculatory impairment also exert considerable influence. Moreover, extensive recognition links inflammation to AIS. Inflammatory responses can exacerbate cerebral edema, hinder healing, and lead to an unfavorable prognosis (29, 30). RDW is positively related to plasma inflammatory biomarkers such as C-reactive protein (31, 32), the erythrocyte sedimentation rate (ESR) (33), soluble tumor necrosis factor-*α* (TNF-α) (34), and interleukin-6 (34, 35). An increased RDW, even within the normal limits, may signify an underlying inflammatory condition, potentially intensifying inflammation and worsening results following AIS. Research has indicated that elevated RDWs correlate with adverse functional outcome in AIS patients at discharge and at the three-month follow-up (26, 36). Second, decreased hemoglobin levels indicate a reduced ability for oxygen transport, resulting in inadequate oxygen supply and an energy deficit in the ischemic penumbra (37). Reduced hemoglobin levels can diminish muscle strength, induce cognitive impairment, and increase fatigue, thereby increasing the risk of frailty in older individuals (38). Moreover, anemia can induce the release of inflammatory mediators, including TNF-*α* (30). Bullock et al. reported that inadequate nutritional status and a weakened immune response may adversely affect patient prognosis (39).

While hemoglobin and red cell distribution width exhibit predictive significance in AIS patients, they are influenced by numerous factors. Our investigation revealed no significant associations between Hb or RDW and adverse outcome in AIS patients. Since Hb and RDW combine to form the HRR, it could offer a more stable and effective evaluation than individual Hb or RDW measurements do. HRR objectively indicates inflammatory and microcirculatory conditions, potentially functioning as a superior biomarker. The HRR, derived from routine hematological parameters, provides a practical and cost-effective tool for early risk stratification in older AIS patients, enabling timely and targeted therapeutic interventions in real-world clinical settings. Prior research has indicated that a lower HRR is correlated with unfavorable outcome in multiple malignant conditions (10, 40–44). Recently, the HRR has become a vital predictor of mortality and prognosis in cardiovascular disease patients (45). A study with 1,816 older participants indicated that an elevated HRR in heart failure patients decreased the likelihood of 3-month readmission by approximately 30% (46). Yuan et al. reported a negative correlation between the HRR and the incidence of severe adverse cardiovascular events in older adults with CHD (47). According to Qu et al., there is an inverse relationship between a low HRR (<9.76) and the likelihood of frailty in older people with CHD (48). These findings indicate that the HRR is a better predictor of frailty than the RDW or Hb level (48). Research using the MIMIC-IV database also revealed that a lower HRR (HRR <9.74) was linked to a lower risk of mortality from any cause in people with AIS and atrial fibrillation (19). In patients with AIS receiving intravenous thrombolysis or mechanical thrombectomy, a decreased HRR is correlated with an increased risk of adverse outcome and mortality (49, 50). Lin et al. reported a significant correlation between a decreased HRR and increased mortality risk in older patients suffering from cerebral hemorrhage (51). Our study corroborates prior research and examines the influence of low HRR values on negative outcome in senior AIS patients after 3 months. When the HRR was ≤10.71, we noted a 26% reduction in the likelihood of unfavorable outcome for older AIS patients with each 1-unit increase in the HRR. These findings suggest that a decreased HRR is correlated with poorer outcome than an elevated HRR. We hypothesize that lower HRR may elevate the risk of adverse outcomes in older individuals with AIS. Additional research is needed to substantiate this notion.

Previous studies have reported that hs-CRP is associated with unfavorable outcome in AIS (51, 52), which is consistent with our findings. Additionally, stress-induced hyperglycemia (FPG/HbA1c) has been linked to increased short-and long-term mortality in AIS, as well as early neurological deterioration (53–55). Traditional inflammatory biomarkers, such as WBC and the Neutrophil-to-Lymphocyte Ratio, have also been associated with poor prognosis in AIS (56, 57). However, in our study, no significant correlations were found between FPG/HbA1c or WBC and unfavorable outcome in AIS. In the ROC prediction model, HRR demonstrated the highest AUC, suggesting that this indicator could potentially offer a more comprehensive and stable prognostic marker for AIS outcome.

Our study has numerous strengths: (1) This is the first investigation into the correlation between baseline HRR levels and adverse outcome in older patients with AIS. (2) The research employed empirical data and included a broad, heterogeneous population. The absence of HRR values likely reduced selection bias. (3) Multiple imputations were used to mitigate statistical bias and improve the reliability of the results. (4) We utilized a two-piecewise logistic regression analysis to examine the threshold influence of the HRR on poor outcome.

However, this study has several limitations. First, the study cohort was derived from a single Korean center, which may not fully represent a broader patient population. Variations in healthcare resources, baseline patient characteristics, and treatment strategies across different regions may influence the association between HRR and unfavorable outcomes. Therefore, future studies should be conducted in multicenter and multi-regional settings to validate these findings and enhance their generalizability. Additionally, we only performed Hb and RDW tests at admission and did not repeat them throughout the hospital stay, thus failing to assess their dynamic changes over time. Previous studies recently highlighted that serial assessments of the Alberta Stroke Program Early CT Score (ASPECTS) may be useful predictors of mortality and poor functional outcomes following thrombolytic therapy (49, 58, 59). The development of a novel clinical prediction model integrating established predictors, serial ASPECTS evaluations, and dynamic HRR changes to predict adverse functional outcomes in elderly patients with AIS remains a significant focus for future research. Futhermore, the study’s results were only assessed via the mRS score. Although the mRS extensively evaluates post-stroke performance, it covers only specific aspects of patient rehabilitation. Lastly, comorbid conditions such as heart failure, chronic kidney disease, malnutrition, iron deficiency, vitamin B12 deficiency, folate deficiency, anemia of chronic disease, and thalassemia can significantly impact red blood cell production and lifespan. These effects may alter hemoglobin (Hb) levels and red cell distribution width (RDW), which are critical hematological parameters. Additionally, thrombolysis and endovascular thrombectomy are essential therapeutic interventions that can substantially influence post-stroke outcomes. However, as this study is based on secondary data analysis, the dataset provided by the original authors did not include these variables, limiting our ability to adjust for potential confounding factors. Future multi-center, prospective studies incorporating these variables are necessary to further validate and refine the observed.

## 5 Conclusion

This study demonstrated a nonlinear association between the HRR and adverse functional outcome in older individuals with AIS. We identified a substantial inverse correlation between elevated HRR and adverse outcome in older people with AIS when the HRR was less than 10.71. Consequently, the HRR predicts favorable clinical outcome in older patients with AIS and may function as an accessible and affordable prognostic biomarker. Timely care for older patients with increased HRR may mitigate adverse AIS outcome.

## Data Availability

The datasets presented in this study can be found in online repositories. The names of the repository/repositories and accession number(s) can be found in the article/Supplementary material.

## Footnotes

^1^ http://www.r-project.org

^2^ www.empowerstats.net
