# Supplementary material for: Association between the hemoglobin-to-red cell distribution width ratio and three-month unfavorable outcome in older acute ischemic stroke patients: a prospective study
Source: Front Neurol. 2025 Mar 11;16:1534564. doi: 10.3389/fneur.2025.1534564 (PMC11963698; doi:10.3389/fneur.2025.1534564)
Supplement: Supplementary file 1 [file Supplementary_file_1.docx]

| **Supplementary Table 1** Details of missing values | | | | | | | | | |
| --- | --- | --- | --- | --- | --- | --- | --- | --- | --- |
| Variables | | The number of missing values | | | The percent of missing values (%) | | | | |
| WBC | | 1 | | | 0.07% | | | | |
| LDL-C | | 57 | | | 3.87% | | | | |
| FBG | | 109 | | | 7.41% | | | | |
| ALT | | 1 | | | 0.07% | | | | |
| Previous mRs | | 1 | | | 0.07% | | | | |
| Abbreviations: WBC, white blood count; LDL-C, low-density lipoprotein cholesterol; FBG, fasting blood glucose; ALT, alanine aminotransferase; mRs, modified Rankin scale. | | | | | | | | | |
|  |  |  |  |  |  |  |  |  |  |
|  |  |  |  |  |  |  |  |  |  |
| **Supplementary Table 2** Baseline characteristics of the participants | | | | | | | | | |
| **Variables** | | **HRR quartile** | | | | | | | ***p-*value** |
| **N** | | **Total (n=1470)** | | **Q1 (<8.96) (n=367)** | **Q2 (8.96–10.30) (n=364)** | | **Q3 (10.31–11.30) (n=370)** | **Q4 (>11.30) (n=369)** |  |
| **Sex, n (%)** | |  | |  |  | |  |  | <0.001 |
| Male | | 854 (58.10%) | | 166 (45.23%) | 172 (47.25%) | | 220 (59.46%) | 296 (80.22%) |  |
| Female | | 616 (41.90%) | | 201 (54.77%) | 192 (52.75%) | | 150 (40.54%) | 73 (19.78%) |  |
| **Age (years), n (%)** | |  | |  |  | |  |  | <0.001 |
| 60 to ＜70 | | 505 (34.35%) | | 86 (23.43%) | 97 (26.65%) | | 156 (42.16%) | 166 (44.99%) |  |
| 70 to＜80 | | 670 (45.58%) | | 184 (50.14%) | 175 (48.08%) | | 156 (42.16%) | 155 (42.01%) |  |
| ≥ 80 | | 295 (20.07%) | | 97 (26.43%) | 92 (25.27%) | | 58 (15.68%) | 48 (13.01%) |  |
| WBC (10^9/L) | | 8.04 ± 2.88 | | 7.97 ± 3.50 | 7.92 ± 2.59 | | 7.90 ± 2.64 | 8.36 ± 2.65 | 0.003 |
| RBC(10^9/L) | | 4.24 ± 0.63 | | 3.55 ± 0.56 | 4.14 ± 0.36 | | 4.44 ± 0.32 | 4.83 ± 0.38 | <0.001 |
| Hb(g/L) | | 132.52 ± 19.62 | | 107.78 ± 14.93 | 129.02 ± 8.12 | | 139.87 ± 7.68 | 153.20 ± 9.25 | <0.001 |
| RDW (%) | | 13.46 ± 1.54 | | 14.88 ± 2.28 | 13.31 ± 0.74 | | 13.01 ± 0.64 | 12.63 ± 0.61 | <0.001 |
| HRR | | 10.00 ± 1.91 | | 7.39 ± 1.34 | 9.69 ± 0.37 | | 10.75 ± 0.30 | 12.14 ± 0.69 | <0.001 |
| PLT (10^9/L) | | 219.04 ± 69.99 | | 220.15 ± 94.47 | 217.16 ± 63.80 | | 216.87 ± 58.90 | 221.96 ± 56.25 | 0.717 |
| LDL-C (mg/dl) | | 103.00 (80.00-128.00) | | 94.00 (70.00-119.25) | 100.00 (78.00-127.25) | | 105.00 (82.00-130.00) | 111.00 (91.00-133.00) | <0.001 |
| BUN (mg/dl) | | 16.00 (13.00-21.00) | | 18.00 (14.00-26.50) | 16.00 (13.00-21.00) | | 15.00 (13.00-19.00) | 15.00 (13.00-19.00) | <0.001 |
| Scr (mg/dl) | | 0.90 (0.74-1.11) | | 0.98 (0.74-1.45) | 0.87 (0.73-1.06) | | 0.84 (0.72-1.03) | 0.93 (0.81-1.09) | <0.001 |
| ALT (U/L) | | 18.00 (13.00-25.00) | | 15.00 (11.00-22.00) | 17.00 (13.00-26.00) | | 18.00 (14.00-24.75) | 20.00 (15.00-29.00) | <0.001 |
| FBG (mmol/L) | | 107.11 ± 37.68 | | 103.11 ± 38.00 | 107.42 ± 37.28 | | 107.61 ± 33.24 | 110.01 ± 41.55 | <0.001 |
| HBA1c (%) | | 6.42 ± 1.11 | | 6.35 ± 0.98 | 6.48 ± 1.05 | | 6.36 ± 1.05 | 6.47 ± 1.32 | 0.17 |
| hs-CRP (mg/L) | | 0.13 (0.04-0.48) | | 0.34 (0.08-2.61) | 0.13 (0.05-0.46) | | 0.07 (0.01-0.21) | 0.12 (0.04-0.31) | <0.001 |
| FIB (mg/L) | | 332.93 ± 92.03 | | 356.96 ± 121.41 | 332.64 ± 86.97 | | 319.86 ± 66.65 | 322.41 ± 79.82 | <0.001 |
| BMI (kg/m^2^) | | 23.22 ± 3.13 | | 22.07 ± 3.30 | 23.16 ± 3.00 | | 23.63 ± 3.10 | 24.01 ± 2.75 | <0.001 |
| **Hypertension, n (%)** | |  | |  |  | |  |  | 0.449 |
| No | | 464 (31.56%) | | 113 (30.79%) | 108 (29.67%) | | 129 (34.86%) | 114 (30.89%) |  |
| Yes | | 1006 (68.44%) | | 254 (69.21%) | 256 (70.33%) | | 241 (65.14%) | 255 (69.11%) |  |
| **DM, n (%)** | |  | |  |  | |  |  | <0.001 |
| No | | 966 (65.71%) | | 225 (61.31%) | 221 (60.71%) | | 250 (67.57%) | 270 (73.17%) |  |
| Yes | | 504 (34.29%) | | 142 (38.69%) | 143 (39.29%) | | 120 (32.43%) | 99 (26.83%) |  |
| **Hyperlipidemia, n (%)** | |  | |  |  | |  |  | 0.019 |
| No | | 948 (64.49%) | | 261 (71.12%) | 229 (62.91%) | | 234 (63.24%) | 224 (60.70%) |  |
| Yes | | 522 (35.51%) | | 106 (28.88%) | 135 (37.09%) | | 136 (36.76%) | 145 (39.30%) |  |
| **Smoking status, n (%)** | |  | |  |  | |  |  | <0.001 |
| No | | 957 (65.10%) | | 274 (74.66%) | 257 (70.60%) | | 235 (63.51%) | 191 (51.76%) |  |
| Yes | | 513 (34.90%) | | 93 (25.34%) | 107 (29.40%) | | 135 (36.49%) | 178 (48.24%) |  |
| **AF, n (%)** | |  | |  |  | |  |  | 0.497 |
| No | | 1099 (74.76%) | | 266 (72.48%) | 268 (73.63%) | | 283 (76.49%) | 282 (76.42%) |  |
| Yes | | 371 (25.24%) | | 101 (27.52%) | 96 (26.37%) | | 87 (23.51%) | 87 (23.58%) |  |
| **CHD, n (%)** | |  | |  |  | |  |  | 0.101 |
| No | | 1275 (86.73%) | | 312 (85.01%) | 306 (84.07%) | | 329 (88.92%) | 328 (88.89%) |  |
| Yes | | 195 (13.27%) | | 55 (14.99%) | 58 (15.93%) | | 41 (11.08%) | 41 (11.11%) |  |
| **NIHSS score at admission, n (%)** | |  | |  |  | |  |  | <0.001 |
| ≤5 | | 971 (66.05%) | | 209 (56.95%) | 244 (67.03%) | | 261 (70.54%) | 257 (69.65%) |  |
| 5 to ≤13 | | 316 (21.50%) | | 89 (24.25%) | 75 (20.60%) | | 79 (21.35%) | 73 (19.78%) |  |
| ＞13 | | 183 (12.45%) | | 69 (18.80%) | 45 (12.36%) | | 30 (8.11%) | 39 (10.57%) |  |
| **Stroke etiology, n (%)** | |  | |  |  | |  |  | <0.001 |
| LAA | | 477 (32.45%) | | 103 (28.07%) | 118 (32.42%) | | 124 (33.51%) | 132 (35.77%) |  |
| SVO | | 288 (19.59%) | | 50 (13.62%) | 71 (19.51%) | | 93 (25.14%) | 74 (20.05%) |  |
| CE | | 427 (29.05%) | | 116 (31.61%) | 111 (30.49%) | | 97 (26.22%) | 103 (27.91%) |  |
| Other determined | | 98 (6.67%) | | 59 (16.08%) | 25 (6.87%) | | 5 (1.35%) | 9 (2.44%) |  |
| Undetermined | | 180 (12.24%) | | 39 (10.63%) | 39 (10.71%) | | 51 (13.78%) | 51 (13.82%) |  |
| **mRs score at admission, n (%)** | |  | |  |  | |  |  | <0.001 |
| ≤2 | | 1008 (68.57%) | | 206 (56.13%) | 249 (68.41%) | | 277 (74.86%) | 276 (74.80%) |  |
| ≥3 | | 462 (31.43%) | | 161 (43.87%) | 115 (31.59%) | | 93 (25.14%) | 93 (25.20%) |  |
| The values are the means ± standard deviations, medians (quartiles) or numbers (%) WBC, white blood count; RBC, red blood cell; Hb, hemoglobin; RDW, red cell volume distribution width; HRR, hemoglobin concentration-to-red cell volume distribution width; PLT, platelet; LDL-C, low-density lipoprotein cholesterol; BUN, blood urea nitrogen; Scr, serum creatinine; ALT, alanine aminotransferase; FBG, fasting blood glucose; HbA1c, hemoglobin A1c, hs-CRP, hypersensitive-reaction protein; FIB, fibrinogen; BMI, body mass index; DM, diabetes mellitus; AF, atrial fibrillation, CHD, coronary heart disease. LAA, large artery atherosclerosis; SVO, small vessel occlusion; CE, cardiac embolism; mRs, modified Rankin scale; NIHSS, National Institute of Health Stroke Scale. | | | | | | | | | |
|  |  |  |  |  |  |  |  |  |  |

| **Supplementary Table 3** The baseline clinical characteristics of elderly patients with AIS. | | | |
| --- | --- | --- | --- |
| **Variables** | **Favorable outcome (n=1008)** | **Unfavorable outcome (n=462)** | ***p*-value** |
| **Sex, n (%)** |  |  | <0.001 |
| Male | 626 (62.10%) | 228 (49.35%) |  |
| Female | 382 (37.90%) | 234 (50.65%) |  |
| **Age (years), n (%)** |  |  | <0.001 |
| 60 to ＜70 | 396 (39.29%) | 109 (23.59%) |  |
| 70 to＜80 | 461 (45.73%) | 209 (45.24%) |  |
| ≥ 80 | 151 (14.98%) | 144 (31.17%) |  |
| WBC (10^9/L) | 7.86 ± 2.64 | 8.43 ± 3.31 | <0.001 |
| RBC(10^9/L) | 4.30 ± 0.59 | 4.12 ± 0.69 | <0.001 |
| Hb(g/L) | 134.67 ± 18.21 | 127.82 ± 21.70 | <0.001 |
| RDW (%) | 13.30 ± 1.26 | 13.79 ± 1.97 | <0.001 |
| HRR | 10.23 ± 1.74 | 9.49 ± 2.14 | <0.001 |
| LDL-C (mg/dl) | 103.00 (81.00-128.00) | 102.00 (74.00-128.00) | 0.337 |
| BUN (mg/dl) | 16.00 (13.00-20.00) | 17.00 (13.00-21.00) | 0.042 |
| Scr (mg/dl) | 0.91 (0.76-1.10) | 0.87 (0.70-1.12) | 0.559 |
| ALT (U/L) | 18.00 (14.00-26.00) | 17.00 (11.00-25.00) | 0.055 |
| FBG (mmol/L) | 103.74 ± 32.48 | 114.95 ± 46.76 | <0.001 |
| HBA1c (%) | 6.39 ± 1.05 | 6.48 ± 1.23 | 0.161 |
| hs-CRP (mg/L) | 0.10 (0.03-0.32) | 0.26 (0.07-1.82) | <0.001 |
| FIB (mg/L) | 325.42 ± 86.90 | 349.30 ± 100.49 | <0.001 |
| BMI (kg/m2) | 23.49 ± 2.97 | 22.65 ± 3.37 | <0.001 |
| **Hypertension, n (%)** |  |  | 0.031 |
| No | 336 (33.33%) | 128 (27.71%) |  |
| Yes | 672 (66.67%) | 334 (72.29%) |  |
| **DM, n (%)** |  |  | 0.028 |
| No | 681 (67.56%) | 285 (61.69%) |  |
| Yes | 327 (32.44%) | 177 (38.31%) |  |
| **Hyperlipidemia, n (%)** |  |  | 0.125 |
| No | 637 (63.19%) | 311 (67.32%) |  |
| Yes | 371 (36.81%) | 151 (32.68%) |  |
| **Smoking status, n (%)** |  |  | <0.001 |
| No | 621 (61.61%) | 336 (72.73%) |  |
| Yes | 387 (38.39%) | 126 (27.27%) |  |
| **AF, n (%)** |  |  | <0.001 |
| No | 791 (78.47%) | 308 (66.67%) |  |
| Yes | 217 (21.53%) | 154 (33.33%) |  |
| **CHD, n (%)** |  |  | 0.478 |
| No | 870 (86.31%) | 405 (87.66%) |  |
| Yes | 138 (13.69%) | 57 (12.34%) |  |
| **NIHSS score at admission, n (%)** |  |  | <0.001 |
| ≤5 | 819 (81.25%) | 152 (32.90%) |  |
| 5 to ≤13 | 144 (14.29%) | 172 (37.23%) |  |
| ＞13 | 45 (4.46%) | 138 (29.87%) |  |
| **Stroke etiology, n (%)** |  |  | <0.001 |
| LAA | 339 (33.63%) | 138 (29.87%) |  |
| SVO | 228 (22.62%) | 60 (12.99%) |  |
| CE | 266 (26.39%) | 161 (34.85%) |  |
| Other determined | 49 (4.86%) | 49 (10.61%) |  |
| Undetermined | 126 (12.50%) | 54 (11.69%) |  |
| The values are the means ± standard deviations, medians (quartiles) or numbers (%) WBC, white blood count; RBC, red blood cell; Hb, hemoglobin; RDW, red cell volume distribution width; HRR, hemoglobin concentration-to-red cell volume distribution width; LDL-C, low-density lipoprotein cholesterol; BUN, blood urea nitrogen; Scr, serum creatinine; ALT, alanine aminotransferase; FBG, fasting blood glucose; HbA1c, hemoglobin A1c, hs-CRP, hypersensitive-reaction protein; FIB, fibrinogen; BMI, body mass index; DM, diabetes mellitus; AF, atrial fibrillation, CHD, coronary heart disease. LAA, large artery atherosclerosis; SVO, small vessel occlusion; CE, cardiac embolism; NIHSS, National Institute of Health Stroke Scale. | | | |

| **Supplementary Table 4** Influencing factors of unfavorable outcomes in AIS using univariate regression analysis | | |
| --- | --- | --- |
| **Variables** | **OR (95 CI%)** | ***p*-value** |
| **Sex** |  |  |
| Male | Ref |  |
| Female | 1.68 (1.35, 2.10) | <0.001 |
| **Age (years)** |  |  |
| 60 to ＜70 | Ref |  |
| 70 to＜80 | 1.65 (1.26, 2.15) | <0.001 |
| ≥ 80 | 3.46 (2.54, 4.73) | <0.001 |
| **BMI (kg/m2)** |  |  |
| <25 | Ref |  |
| 25-29.9 | 0.65 (0.50, 0.86) | 0.002 |
| >30 | 1.77 (0.87, 3.59) | 0.112 |
| WBC | 1.07 (1.03, 1.11) | <0.001 |
| RBC | 0.63 (0.53, 0.75) | <0.001 |
| Hb | 0.98 (0.98, 0.99) | <0.001 |
| RDW | 1.22 (1.13, 1.32) | <0.001 |
| HRR | 0.82 (0.77, 0.87) | <0.001 |
| PLT | 1.00 (1.00, 1.00) | 0.949 |
| LDL-C | 1.00 (1.00, 1.00) | 0.337 |
| BUN | 1.01 (1.00, 1.02) | 0.042 |
| Scr | 1.03 (0.93, 1.14) | 0.560 |
| ALT | 0.99 (0.98, 1.00) | 0.057 |
| FBG | 1.01 (1.00, 1.01) | <0.001 |
| hs-CRP | 1.14 (1.09, 1.18) | <0.001 |
| HBA1c | 1.08 (0.97, 1.21) | 0.162 |
| **Hypertension** |  |  |
| No | Ref |  |
| Yes | 1.30 (1.02, 1.66) | 0.031 |
| **DM** |  |  |
| No | Ref |  |
| Yes | 1.29 (1.03, 1.63) | 0.028 |
| **Hyperlipidemia** |  |  |
| No | Ref |  |
| Yes | 0.83 (0.66, 1.05) | 0.126 |
| **Smoking status** |  |  |
| No | Ref |  |
| Yes | 0.60 (0.47, 0.77) | <0.001 |
| **CHD, n (%)** |  |  |
| No | Ref |  |
| Yes | 0.89 (0.64, 1.23) | 0.478 |
| **AF** |  |  |
| No | Ref |  |
| Yes | 1.82 (1.43, 2.33) | <0.001 |
| **Stroke etiology** |  |  |
| LAA | Ref |  |
| SVO | 0.65 (0.46, 0.91) | 0.014 |
| CE | 1.49 (1.13, 1.96) | 0.005 |
| Other determined | 2.46 (1.58, 3.82) | <0.001 |
| Undetermined | 1.05 (0.72, 1.53) | 0.788 |
| Abbreviations:BMI, body mass index; WBC, white blood count; RBC, red blood cell; Hb, hemoglobin; RDW, red cell volume distribution width; HRR, hemoglobin concentration-to-red cell volume distribution width; LDL-C, low-density lipoprotein cholesterol; BUN, blood urea nitrogen; Scr, serum creatinine; ALT, alanine aminotransferase; FBG, fasting blood glucose; HbA1c, hemoglobin A1c, hs-CRP, hypersensitive-reaction protein; DM, diabetes mellitus; AF, atrial fibrillation; CHD, coronary heart disease. LAA, large artery atherosclerosis; SVO, small vessel occlusion; CE, cardiac embolism; NIHSS, National Institute of Health Stroke Scale. | | |

| **Supplementary Table 5** Relationship between Hb and unfavorable outcome 3 months after AIS in different models | | | | | | |
| --- | --- | --- | --- | --- | --- | --- |
| **Variable** | **Crude model (OR,95%CI)** | ***p-*value** | **Model I (OR,95%CI)** | ***p-*value** | **Model II (OR,95%CI)** | ***p-*value** |
| Hb (g/L) | 0.98 (0.98, 0.99) | <0.001 | 0.99 (0.98, 0.99) | <0.001 | 0.98 (0.96, 0.99) | 0.005 |
| Hb (g/L) (Quartiles) |  |  |  |  |  |  |
| Q1 (<120) | Ref |  | Ref |  | Ref |  |
| Q2 (120-133) | 0.59 (0.43, 0.80) | <0.001 | 0.59 (0.43, 0.82) | 0.001 | 0.67 (0.43, 1.03) | 0.068 |
| Q3 (134 - 145) | 0.47 (0.34, 0.64) | <0.001 | 0.55 (0.40, 0.77) | <0.001 | 0.58 (0.34, 0.99) | 0.047 |
| Q4 (>145) | 0.44 (0.32, 0.60) | <0.001 | 0.59 (0.42, 0.83) | 0.0021 | 0.57 (0.29, 1.10) | 0.094 |
| *p* for trend | 0.98 (0.97, 0.99) | <0.001 | 0.99 (0.98, 0.99) | 0.0007 | 0.99 (0.97, 1.00) | 0.082 |
| Crude mode 1: we did not adjust for other covariates; Model I: we adjusted for sex and age; Model II: Model I + WBC, RBC, PLT, LDL-C, BUN, FBG, ALT, hs-CRP, BMI, hypertension, DM, hyperlipidemia, CHD, AF, smoking status, NIHSS score at admission, and previous mRs. | | | | | | |
|  |  |  |  |  |  |  |
|  |  |  |  |  |  |  |

| **Supplementary Table 6** Relationship between RDW and unfavorable outcome 3 months after AIS in different models | | | | | | |
| --- | --- | --- | --- | --- | --- | --- |
| **Variable** | **Crude model (OR,95%CI)** | ***p-***value | **Model I (OR,95%CI)** | ***p-***value | **Model II (OR,95%CI)** | ***p-***value |
| RDW (%) | 1.22 (1.13, 1.32) | <0.001 | 1.21 (1.12, 1.31) | <0.001 | 1.18 (1.07, 1.29) | <0.001 |
| RDW (%) (Quartiles) |  |  |  |  |  |  |
| Q1 (<12.6) | Ref |  | Ref |  | Ref |  |
| Q2 (12.6-13.1) | 0.93 (0.67, 1.29) | 0.667 | 0.89 (0.63, 1.24) | 0.485 | 0.83 (0.56, 1.21) | 0.331 |
| Q3 (13.2 - 13.7) | 1.11 (0.79, 1.54) | 0.552 | 1.01 (0.72, 1.42) | 0.948 | 1.04 (0.71, 1.53) | 0.841 |
| Q4 (>13.7) | 1.67 (1.22, 2.29) | 0.001 | 1.55 (1.12, 2.14) | 0.008 | 1.35 (0.91, 1.98) | 0.133 |
| *p* for trend | 1.29 (1.13, 1.48) | <0.001 | 1.26 (1.10, 1.44) | 0.001 | 1.19 (1.01, 1.40) | 0.041 |
| Crude mode 1: we did not adjust for other covariates; Model I: we adjusted for sex and age; Model II: Model I + WBC, RBC, PLT, LDL-C, BUN, FBG, ALT, hs-CRP, BMI, hypertension, DM, hyperlipidemia, CHD, AF, smoking status, NIHSS score at admission, and previous mRs. | | | | | | |
|  |  |  |  |  |  |  |
|  |  |  |  |  |  |  |

| **Supplementary Table 7** Relationship between WBC and unfavorable outcome 3 months after AIS in different models | | | | | | |
| --- | --- | --- | --- | --- | --- | --- |
| **Variable** | **Crude model (OR,95%CI)** | ***p-***value | **Model I (OR,95%CI)** | ***p-***value | **Model II (OR,95%CI)** | ***p-***value |
| WBC (10⁹/L) | 1.07 (1.03, 1.11) | <0.001 | 1.07 (1.03, 1.11) | 0.001 | 1.02 (0.97, 1.07) | 0.556 |
| WBC (10⁹/L) (Quartiles) |  |  |  |  |  |  |
| Q1 (<6.15) | Ref |  | Ref |  | Ref |  |
| Q2 (6.15-7.53) | 1.13 (0.82, 1.56) | 0.467 | 1.18(0.85, 1.65) | 0.326 | 1.16 (0.77, 1.75) | 0.478 |
| Q3 (7.54 - 9.27) | 1.27 (0.93, 1.75) | 0.137 | 1.34 (0.97, 1.85) | 0.080 | 1.31 (0.82, 2.11) | 0.261 |
| Q4 (>9.27) | 1.60(1.17, 2.19) | 0.003 | 1.71(1.24, 2.36) | 0.001 | 1.31 (0.65, 2.66) | 0.455 |
| *p* for trend | 1.08 (1.03, 1.14) | 0.002 | 1.10 (1.04, 1.15) | <0.001 | 1.05 (0.93, 1.18) | 0.433 |
| Crude mode 1: we did not adjust for other covariates; Model I: we adjusted for sex and age; Model II: Model I + WBC, RBC, PLT, LDL-C, BUN, FBG, ALT, hs-CRP, BMI, hypertension, DM, hyperlipidemia, CHD, AF, smoking status, NIHSS score at admission, and previous mRs. | | | | | | |
|  |  |  |  |  |  |  |
|  |  |  |  |  |  |  |
| **Supplementary Table 8** Relationship between hs-CRP and unfavorable outcome 3 months after AIS in different models | | | | | | |
| **Variable** | **Crude model (OR,95%CI)** | ***p-***value | **Model I (OR,95%CI)** | ***p-***value | **Model II (OR,95%CI)** | ***p-***value |
| hs-CRP (mg/L) | 1.14 (1.09, 1.18) | <0.001 | 1.12 (1.08, 1.17) | <0.001 | 1.10 (1.05, 1.16) | <0.001 |
| hs-CRP (mg/L) (Quartiles) |  |  |  |  |  |  |
| Q1 (<0.04) | Ref |  | Ref |  | Ref |  |
| Q2 (0.04-0.12) | 0.94 (0.66, 1.33) | 0.722 | 0.95 (0.66, 1.35) | 0.764 | 1.11 (0.75, 1.65) | 0.609 |
| Q3 (0.13 - 0.47) | 1.44 (1.03, 2.01) | 0.034 | 1.37 (0.97, 1.92) | 0.073 | 1.31 (0.90, 1.91) | 0.165 |
| Q4 (>0.48) | 3.40 (2.46, 4.71) | <0.001 | 3.18 (2.28, 4.43) | <0.001 | 2.22 (1.43, 3.44) | <0.001 |
| *p* for trend | 1.85 (1.62, 2.12) | <0.001 | 1.80 (1.57, 2.06) | <0.001 | 1.45 (1.19, 1.76) | <0.001 |
| Crude mode 1: we did not adjust for other covariates; Model I: we adjusted for sex and age; Model II: Model I + WBC, RBC, PLT, LDL-C, BUN, FBG, ALT, hs-CRP, BMI, hypertension, DM, hyperlipidemia, CHD, AF, smoking status, NIHSS score at admission, and previous mRs. | | | | | | |
|  |  |  |  |  |  |  |
|  |  |  |  |  |  |  |

| **S9 Table** Relationship between FPG/HbA1c and unfavorable outcome 3 months after AIS in different models | | | | | | |
| --- | --- | --- | --- | --- | --- | --- |
| **Variable** | **Crude model (OR,95%CI)** | ***p-***value | **Model I (OR,95%CI)** | ***p-***value | **Model II (OR,95%CI)** | ***p-***value |
| FPG/HbA1c | 1.08 (1.05, 1.11) | <0.001 | 1.09 (1.06, 1.12) | <0.001 | 1.05 (0.99, 1.12) | 0.111 |
| FPG/HbA1c (quartiles) |  |  |  |  |  |  |
| Q1 (<14.33) | Ref |  | Ref |  | Ref |  |
| Q2 (14.33-16.44) | 0.72 (0.49, 1.06) | 0.095 | 0.76 (0.51, 1.14) | 0.186 | 0.63 (0.40, 0.99) | 0.046 |
| Q3 (16.45 - 19.05) | 1.03 (0.71, 1.49) | 0.871 | 1.14 (0.78, 1.67) | 0.494 | 0.80 (0.51 1.26) | 0.336 |
| Q4 (>19.06) | 1.94 (1.36, 2.77) | <0.001 | 2.25 (1.56, 3.25) | <0.001 | 1.33 (0.76, 2.35) | 0.320 |
| p for trend | 1.092 (1.05, 1.13) | <0.001 | 1.11 (1.07, 1.15) | <0.001 | 1.04 (0.98, 1.11) | 0.224 |
| Crude mode 1: we did not adjust for other covariates; Model I: we adjusted for sex and age; Model II: Model I + WBC, RBC, PLT, LDL-C, BUN, FBG, ALT, hs-CRP, BMI, hypertension, DM, hyperlipidemia, CHD, AF, smoking status, NIHSS score at admission, and previous mRs. | | | | | | |
|  |  |  |  |  |  |  |
|  |  |  |  |  |  |  |

**
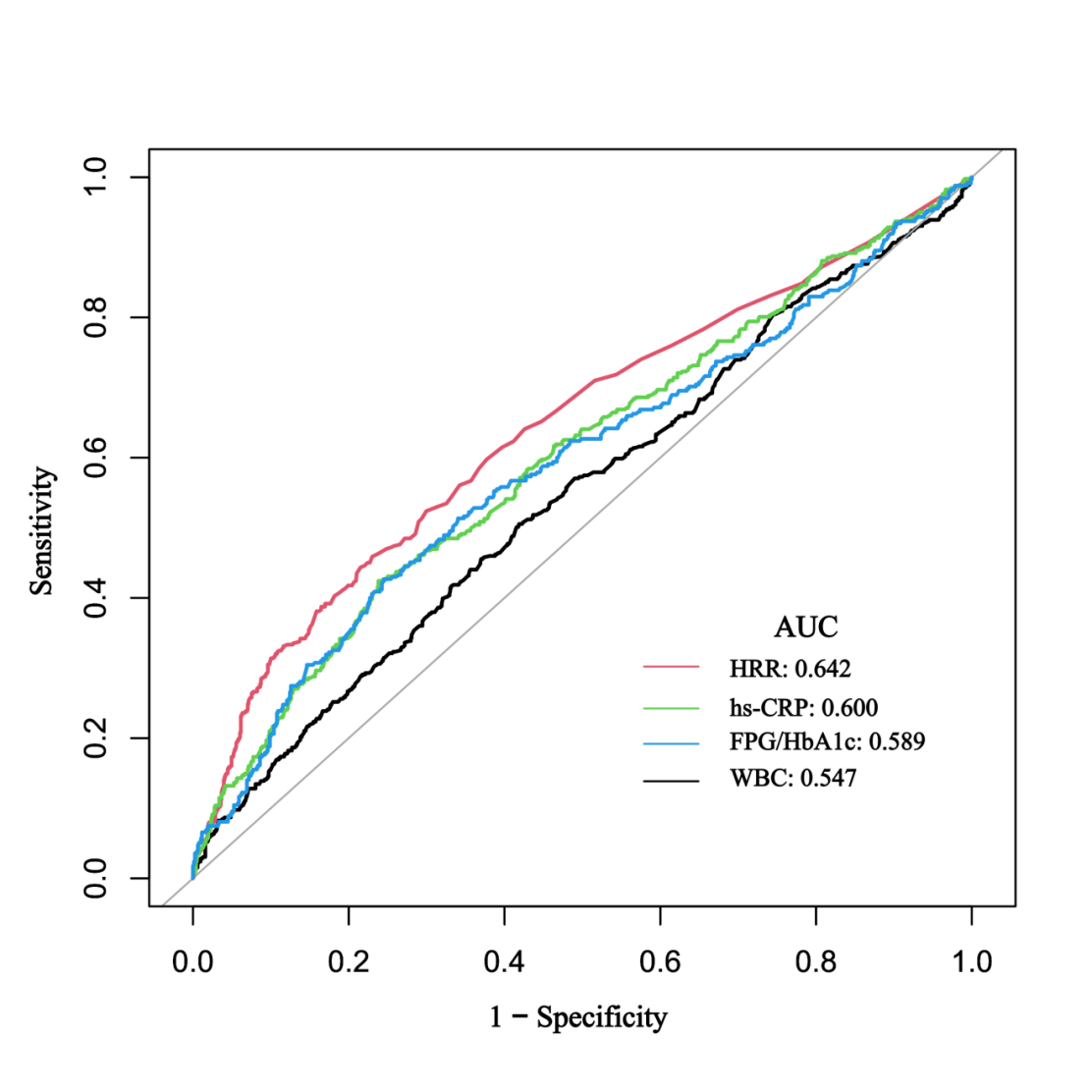
**

**Supplementary Figure 1** Receiver operating characteristic (ROC) curves for HRR, hs-CRP, FPG/HbA1c, and WBC in predicting 3-month unfavorable outcome in older adults with AIS.
